# Video Head Impulse Test Changes Related to Obstructive Sleep Apnea: In Reply to the Work of Xin-Da Xu et al.

**DOI:** 10.3389/fneur.2022.889187

**Published:** 2022-04-28

**Authors:** Alessandro Micarelli, Andrea Viziano, Marco Alessandrini

**Affiliations:** ^1^ITER Center for Balance and Rehabilitation Research (ICBRR), Rome, Italy; ^2^Institute of Mountain Emergency Medicine, Eurac Research, Bolzano, Italy; ^3^Department of Clinical Sciences and Translational Medicine, University of Rome Tor Vergata, Rome, Italy

**Keywords:** video head impulse test (vHIT), obstructive sleep apnea, vestibular hypofunction, continuous positive air pressure therapy (C-PAP), vestibular nuclei

We read with interest the last issue of your Journal in which the following article has been published by Xin-Da Xu et al.: “Uneven Effects of Sleep Apnea on Semicircular Canals and Otolithic Organs” (1). In their valuable work, the authors discussed different works depicting changes in vestibular evoked myogenic potentials and caloric testing in patients with obstructive sleep apnea (OSA). However, they highlighted that only a few works focused on semicircular canal weakness in patients affected by OSA when studied by means of video head impulse test (vHIT) and pointed attention to the lack of control groups in previous works [see for example Birk et al. (2)]. Although we agree that few published works exist in this field, we believe that the researchers interested in the topic may appreciate that one previous study not only found a vestibulo-ocular reflex (VOR) gain deficit in patients with OSA, when studied by means of vHIT and compared with a group of healthy subjects, but this finding was significantly related to changes in oxygen saturation (3). To corroborate such relationships, a further study by Alessandrini et al. demonstrated a significant improvement in VOR gain after 1 year of continuous positive airway pressure treatment in the same sample of participants with OSA (4). Furthermore, an interesting debate arose in 2018 in an issue of Sleep Medicine Reviews between the research groups of Besnard S and Alessandrini M. They finally assessed that beyond the impaired higher-level vestibular neural inflow related to sleep deprivation, the functional alterations of the vestibular nuclei may be an indirect indicator of abnormal activity of the respiratory nuclei during OSA, considering their anatomical contiguity and the susceptibility of the posterior labyrinth to a hypoxic state (5–7). In this scenario, we believe that although more studies are needed to better understand vHIT changes in the course of the natural history of OSA, a certain degree of evidence has been already obtained and we believe that it could be helpful to integrate the work of Xin-Da Xu et al. with these notions.

## Author Contributions

AM, AV, and MA wrote the manuscript. All authors contributed to the article and approved the submitted version.

## Conflict of Interest

The authors declare that the research was conducted in the absence of any commercial or financial relationships that could be construed as a potential conflict of interest.

## Publisher's Note

All claims expressed in this article are solely those of the authors and do not necessarily represent those of their affiliated organizations, or those of the publisher, the editors and the reviewers. Any product that may be evaluated in this article, or claim that may be made by its manufacturer, is not guaranteed or endorsed by the publisher.
